# Experiences of patients with chronic obstructive pulmonary disease receiving integrated telehealth nursing services during COVID-19 lockdown

**DOI:** 10.1186/s12912-022-00967-2

**Published:** 2022-08-01

**Authors:** Antonia Arnaert, Hamza Ahmad, Shameera Mohamed, Emilie Hudson, Stephanie Craciunas, Alice Girard, Zoumanan Debe, Joséphine Lemy Dantica, Candice Denoncourt, Geneviève Côté-Leblanc

**Affiliations:** 1 Ingram School of Nursing, McGill University, 680 Sherbrooke West, Montreal, Quebec H3A 2M7 Canada; 2 Montreal West Island Integrated University Health and Social Service Centre, Montreal, Canada

**Keywords:** COVID-19 pandemic, Integrated telemonitoring, Telehealth nursing, Chronic obstructive pulmonary disease, Chronic disease management, Self-management, Primary care, Qualitative research

## Abstract

**Objective:**

Even though telemonitoring existed prior to COVID-19, the need was accelerated for patients with COPD due to the limited medical consultations and the anxiety for being infected. To our knowledge, no qualitative study has explored the experiences of COPD patients and the potential benefits of receiving integrated telehealth nursing services during the lockdown.

**Methods:**

Ten participants were interviewed after receiving telehealth nursing services for 3 months; data was analyzed thematically.

**Results:**

Irrespective of COPD severity, all participants expressed that the existing fragmented follow-up care was intensified during COVID. The telenursing services provided them with the comfort and reassurance that a clinician is available for support, advice, and care during the times of isolation. Despite diverse opinions regarding the respiratory-related questions for follow-up, all were enthusiastic about the use of the oximeter in their daily lives. None perceived sharing data as an issue, instead they encouraged the exchange across levels of care.

**Conclusion:**

Despite an appreciation for the service, it is imperative for sustainability reasons that standardized protocols are developed to balance patient preferences in terms of response frequency and the clinical data needed for a telenurse to provide appropriate interventions.

## Background

Chronic obstructive pulmonary disease (COPD) is a progressive, debilitating illness that affects a projected number of 594,000 people in the province of Quebec, Canada [1]. It is believed that this estimate is higher as 60–85% of people live with the condition without knowing. This disease, when compared to other chronic conditions, has the highest rate of hospital readmissions (19.4%) [2]. Unfortunately, many patients with this disease resort to episodic emergency care to cope with poorly managed symptoms of breathlessness [3], with an average incidence rate of approximately 1–3 exacerbations annually [4]. Yet, abundant evidence exists that self-management interventions, including a COPD exacerbation action plan, are associated with improved patients’ quality of life, knowledge of disease and self-management skills, and lower risk of respiratory-related hospital admissions and emergency visits [5–7]. When these self-management strategies are combined with telemonitoring, the effect is potentiated, allowing for the provision of better supportive care.

Telemonitoring which uses a variety of information and communication technologies to provide services remotely for patients with co-morbidities has been introduced more than 20 years ago. Although not novel, the intervention is complex and often difficult to evaluate as it may consist of the transmission of patient physiological and other non-invasive data, such as patient’s health symptoms, to a healthcare professional only, or in combination with the telenurse’s offering of tailored responsive services including telephone support, self-management education, and care coordination [8]. This latter form is often called “integrated telemonitoring” [9]. Despite the various modalities of each telenursing intervention, which are often designed for a specific healthcare setting and with different generations of telemonitoring devices and platforms [10], their efficacy may also be impacted by the behavior of the provider delivering the intervention, and the patients receiving the intervention who might fail to comply [11]. Although a ‘one glove fits all’ approach in offering telemonitoring for COPD patients seems too simplistic for such a heterogenous population [10], evidence exists that both interventions, telemonitoring (measuring physiological parameters and symptoms) only and integrated telemonitoring, reduce emergency room (ER) visits significantly, yet the application of integrated telemonitoring is more effective in reducing ER visits, and offers better health outcomes for patients with severe to very severe COPD [9]. Moreover, integrated telemonitoring makes patients aware of their illness, facilitates their natural coping and acceptance of their disease, which may enhance their overall health and well-being [12].

Improving COPD patients’ psychological and mental health was especially important during the COVID-19 lockdown as they experienced isolation, fear of infection due to being a high-risk population and many of their regular medical follow-up consultations were cancelled or replaced by a telephone consultation which minimizes the risk of transmitting the coronavirus [13, 14]. Although limited evidence exists on the remote care delivery for COPD during the pandemic, its expedited implementation and adoption has provided patients with some type of care continuity in a safe manner. However, the predominant use of telephone-only contact with a clinician without the provision of telemonitoring equipment, such as pulse oximeters and spirometers, only allowed for specific planned activities such as gathering patient health information, providing education on smoke cessation and COPD, and developing self-management plans; the full in-home integrated telemonitoring experience for patients was lacking [15]. Hence, in this study we sought to better understand the overall experiences of patients with a primary diagnosis of COPD and without coronavirus symptoms receiving integrated telehealth nursing services during the COVID-19 lockdown. The research questions are: 1) How did patients with COPD perceive the follow-up care of a telehealth nurse during the COVID-19 lockdown? 2) What are the potential benefits of an integrated telemonitoring system for patient’s suffering from COPD?

### Integrated telehealth nursing services

Once patients agreed to participate in the telemonitoring project, the research assistants scheduled a one-hour telephone appointment to install and educate patients on how to use the telemonitoring platform on a mobile device or desktop computer. All patients were provided with a non-invasive pulse oximeter, and those who did not have a mobile device, or their computer was outdated, received a project tablet computer. The AlayaCare telemonitoring platform is a cloud-based application that has the capability of secure, high-quality videoconferencing and remote physiological monitoring, based on input data from patients using peripherals. In this study, COPD patients were asked, according to their individualized care plan, to enter and submit manually to the telenurse, in real time, their clinical data through the platform. More specifically, the protocol required that patients submit both their blood oxygen saturation levels and pulse daily during a 12-months period, and to answer five specific COPD-related questions. Participants could enter their oxygen levels multiple times during the day and night; however, it was required to complete the questions with different answer categories once every day. The following questions were: *How would you describe your level of shortness of breath (SOB) today? How many times did you spit today? What color was your spit today? Was your spit thicker than usual? How often did you cough today?* On a weekly basis they were asked to answer 10 questions in a nominal format regarding their level of physical activity, fatigue, anxiety, sleeping and smoking behavior, emotional well-being, and change in medical prescriptions during the past week. Tailored patient education material was made available on the system to support them and complement their knowledge regarding lifestyle changes. When a measurement of clinical data was outside of expected patient-specific parameters, the telenurse would contact the patient, provide the necessary interventions, and if needed communicate with the interdisciplinary team at the COPD clinic of a local community hospital. The telenurse and members of the COPD team, two pneumologists and one COPD clinic nurse had access to the telemonitoring platform, however, only the clinic nurse used the system in her clinical practice. After each intervention, all clinicians would complete the interdisciplinary notes on the platform to enhance patient information exchange and continuity of care. The telenurse was a community health nurse who received training on the use of the platform, the COPD disease, and management at the clinic.

## Methods

### Design, sample and recruitment

This qualitative descriptive study was part of a larger study that evaluated the use of a telemonitoring system to enhance patient health-related outcomes, and reduce ER visits and hospitalizations, and which took place from June 2020 to November 2021. In June 2020, a sample of 140 patients who had a COPD diagnosis classified as a risk D for severity of airflow obstruction and were followed by the pneumologists at the local community hospital, were selected by the COPD clinic nurse. Research assistants would phone all patients to explain the project and verify if they would be interested to participate. The recruitment of potential participants was done progressively over several months, and by September 2020 we had a purposive sample of 10 participants enrolled, of which 6 were female and 4 were male, and who received telehealth nursing services for approximately 3 months.

Participants had an average age of 71.5 years (ranging from 67 to 80). All participants except two (P1, P9) were francophones, and only one female subject (P10) was employed. Of the 10 participants, 6 (P1, P3, P5–8) were legally married, 2 (P9–10) divorced, 1 (P4) single and 1 person (P2) was a widow. Participants’ level of education varied: 5 (P1, P6–7, P9–10) persons had a secondary degree and 4 (P2–5) had a tertiary degree. Using the staging system of the Global Initiative for Chronic Obstructive Lung Disease (GOLD), which considers a variety of factors, such as exacerbations, symptom severity and FEV_1_, 2 persons (P3, P7) were classified as GOLD grade 4 with ‘very severe COPD’, 4 (P1–2, P6, P9–10) as GOLD grade 3 with ‘severe COPD’, and 2 (P4–5) as GOLD grade 1 ~ 2 with ‘mild to moderate COPD. In terms of the ABCD assessment, all participants were labeled in the GOLD group D, meaning that they experienced more severe symptoms, such as greater dyspnea and/or exercise tolerance. As shown in Table 1, only 2 participants (P1, P3) had no co-morbidities, 8 participants except P6 had been hospitalized in the last 2 years, and 3 persons (P5, P9–10) were heavy smokers; they smoked approximately 1 pack of cigarettes per day. Participants’ level of mobility varied; some (P2–3, P9) had no mobility problems while others used assistive devices (P1, P4, P7, P10) or were limited in their mobility due to shortness of breath or joint pain (P5, P6, P8). All participants had Internet connectivity at home, and a tablet computer was given to 2 persons (P1, P9). Fear of contracting the coronavirus was experienced by all participants, except for P4 and P5, who considered it a reality. They were nervous, anxious, and afraid of being alone and dying.

**Table 1:** Socio-demographic data

**Subject**	**Age**	**Gender**	**Marital Status**	**Employment**	**Education**	**Mother Tongue**	**COPD GOLD**	**Co-Morbidities**	**Date of Last Hospitalization / ER Visit**
P1	71	Female	Married	Retired	Secondary	French	GOLD 3D	COPD	2020
P2	79	Female	Widowed	Retired	Tertiary	English	GOLD 3D	COPD, Hypertension	2019
P3	80	Male	Married	Retired	Tertiary	English	GOLD 4D	COPD	2019
P4	69	Male	Single	Retired	Tertiary	English	GOLD 1/2D	COPD, Hypertension, Eczema	2019
P5	67	Female	Married	Retired	Tertiary	English	GOLD 1/2D	COPD, Arthritis, Insomnia	Unknown
P6	70	Female	Married	Retired	Secondary	English	GOLD 3D	COPD, Cardiac Problems	2014
P7	70	Male	Married	Retired	Secondary	English	GOLD 4D	COPD, Hypertension	2019
P8	71	Female	Married	Retired	Tertiary	English	GOLD 1D	COPD, Hypertension, Cardiac Problems, Hypercholesterolemia, Anaemia, Chronic Constipation	2020
P9	67	Male	Divorced	Retired	Secondary	French	GOLD 3D	COPD, Hypercholesterolemia	2019
P10	71	Female	Divorced	Employed	Secondary	English	GOLD 3D	COPD, Hypertension, Hypercholesterolemia	2020
**Subject**		**COPD Action Plan**	**Smoking Behaviour**	**Mobility**	**Fear Contracting Coronavirus**	**Home Internet**		**Mobile Device / Computer**	
P1		Yes	Quit	Use walker if needed	Yes, afraid to die	Yes		Project Tablet	
P2		Yes	Light	Walk independent 2 x/week	Yes, nervous	Yes		Personal Tablet	
P3		Yes	Quit	No mobility problems	Yes, anxious	Yes		Laptop	
P4		Yes	Light	Walks with cane	No fear	Yes		Intelligent Phone	
P5		Yes	Heavy	Waiting for hip replacement	No, I am simply more cautious	Yes		Intelligent Phone	
P6		Yes	Moderate	Limited mobility due to SOB and ankle pain	Yes, nervous	Yes		Laptop	
P7		Yes	Quit	Use walker if needed	Yes, afraid to die	Yes		Laptop	
P8		Yes	Quit	Moves around with difficulty due to pain	Yes, afraid of being alone	Yes		Personal Tablet	
P9		Yes	Heavy	No mobility problems	Yes, due to his condition	Yes		Intelligent Phone, Project Tablet	
P10		Yes	Heavy	Uses walker, moves around with difficulty	Yes	Yes		Desktop PC	

### Data collection

Semi-structured interviews in English or French, lasting approximately 60 to 90 minutes, were conducted by the first and third authors at a convenient time for the participant. A joint interview was conducted with participants P6–7; a married couple who both are suffering from COPD. Both authors were present for all interviews, which were audio-recorded and conducted over the phone or through ZOOM videoconferencing. The interview guide included questions such as: *Prior to the pandemic, how were you medically followed for your COPD? What were your initial thoughts about receiving telenursing support during the COVID lockdown? What are your experiences so far with the platform? What are the challenges you have encountered using the system? How do you think this telemonitoring system may be helpful to you?* To ensure alignment between the study aim and the interview questions, the guide was pilot-tested and validated with key informants, and further refinements were made after the first few interviews [16]. At the start of each interview, the purpose was re-explained, and a sociodemographic questionnaire was completed at the start of the larger study.

### Data analysis

All interviews were transcribed in the language of the interview and then translated in English for coding purposes. Each transcript was thematically analyzed using the inductive approach, described by Elo and Kyngäs [17], and supplemented with field notes. A process of open coding was used to assign captions to segments of the transcripts. Codes were organized into categories and themes, which captured similar concepts, from which descriptive statements were formed and supported with quotes. This process was repeated until consensus was reached between the first and second authors. In this study, several strategies were used to enhance the credibility of the findings. Although the interviewers have experience in qualitative research methods, they conducted two mock interviews to refine the semi-structured interview protocol which included several prompts that allowed the expansion of answers and the opportunity for requesting more information, for improving time management, and overall running of the interviews. Both techniques, peer debriefing and member checking, were used to avoid possible biases and preconceptions, and to make sure that the participants’ experiences are adequately represented. To address confirmability, dependability and transferability, the interviewers wrote reflective notes immediately after each interview, documented personal feelings, insights, and committed to a detailed description of the research methods, participants, and settings.

### Ethical considerations

Research ethics approval was obtained from the Research Ethics Committee of an Integrated Health and Social Services Centre (SMH #19–11) on August 16, 2019. All participants signed the consent form prior to the start of the larger study, and written information was provided explaining the study purpose, participant involvement, the right to withdraw at any stage, and data confidentiality.

## Results

Despite efforts in our healthcare systems for optimal integration, the first theme *Fragmented Care Further Intensified During COVID* addresses the fragmented care and lack of standardization in terms of follow-up care experienced by participants prior to the pandemic, which has been exacerbated during COVID-19 lockdown. However, this lack of continuity of care experienced by subjects during the increased isolation due to the outbreak was to a certain extent compensated by having access to a telenurse, who is “monitoring me” and provides personalized feedback and advice; which in return reduced participants fear and anxiety. Those experiences are described in the second theme *Knowing Someone is There to Help, Especially in COVID Times.* Interestingly, the importance of having access to and using an oximeter became apparent when participants utilized it to monitor their own progress and develop strategies to cope with their symptoms. In essence the third theme, *Unparalleled Benefits of an Oximeter*, highlighted the participants’ ability to self-manage and integrate their COPD into their daily lives via the regular use of an oximeter. Nonetheless, as discussed by the participants, communication between different healthcare providers is imperative to providing non-fragmented follow-up care. It was evident that the development of an integrated telemonitoring system would not only facilitate a clear communication between different healthcare providers but also help integrate the different domains of healthcare, as described in the fourth theme *Fragmented Levels of Care to Integrated Healthcare Delivery.* Overall, contrary to the common preconceptions regarding telemonitoring, in the last and final theme *Proving Misconceptions Otherwise,* most participants reported having no problems with data sharing and confidentiality, and felt confident in using the technology for telemonitoring purposes.

### Fragmented care further intensified during COVID

When discussing participants’ follow-up care pre-COVID, all felt to some extent that it was impersonal and fragmented. Four participants (P3, P5, P6, P9) indicated receiving no more than two pneumologist visits per year, often with no interim follow-up, medical or nursing, specifically for their COPD. When asked about visiting a COPD specialist, participant P3 stated: “Nobody, I have many other doctors but nobody for my COPD.” Other participants (P1–2) relied on their family doctors for follow-up, saying; “When I have increased problems breathing and my oxygen levels are going down, I am thinking it is time to start my action plan. I call the family doctor and he usually agrees (P2).” Participant P7 added; “My family doctor and specialist have so many people they are responsible for that they cannot get very involved [in my follow-up].” Participants (P4–8) noted that they were not the only patients their doctors have, and as such, their time with them is divided. Participant P8 further explained that there is no set protocol when dealing with a COPD exacerbation; “If I am worried about something, I call the secretary, or my family doctor, or I just go to the emergency room.” Not all participants reported having fragmented follow-up care, however, a lack of standardization was noticed by some participants. For example, participant P7 mentioned having two lung specialists, while participant P10 saw no other doctor besides her general practitioner. Even when participants were able to receive follow-up care from their specialist, some reported having unpleasant experiences. One participant P6 claimed that the physician did not want her as a patient while another P1 reported having an unpleasant altercation with her pneumologist; “She [the pneumologist] said, ‘I was always at the hospital’. I asked her what I should do when it [shortness of breath] happened. I told her ‘give me your phone number so that I could call you’. She got so mad, she cancelled me. I went home and panicked” (P1).

Most participants (P1, P3–4, P7, P9–10) stated coping with their COPD on a “day by day basis” as it often severely limited their ability to perform daily activities and management at home was becoming increasingly difficult. Participant P7 mentioned: “I take it day by day and whatever happens. You might think you can control it, but you cannot because the weather changes, it can be hot, humid, anything. I used to golf a lot and now I cannot, so it changes you that way.” Participant P10 added: “It has gotten worse with the years. Managing it at home is hard. I am not able to do what I used to do, as far as housework and such. Because of COVID, I have stopped going out […] did not do any exercise so that has taken away my energy. My condition has not improved at all since then [COVID]. Due to the COVID isolation, three participants P4, P6 and P9 experienced a smoking relapse, saying: “I went for 4 years without smoking, but when COVID started, I started smoking again” (P4). Despite some participants indicating a worsening of their health due to COVID, participant P5 indicated: “I find since COVID and retirement, I do not get sick as much. I used to use my action plan three times a year, and it has now been a year since I have had to use it. It is probably because I stay home, I do not go out to friends’ homes, and I spend less time going to different stores. So, in a way, COVID has helped”. Overall, within the current COVID-19 context, participants (P1–2, P4, P6, P9–10) were more concerned and anxious about their health due to their vulnerability for severe consequences because of their illness. Participant P3 stated when asked about concerns regarding COVID-19 and COPD: “Of course, I am concerned. I do have a weak immune system, I have had cancer, polyps of my lungs, so I am concerned. I do not do sleepovers or anything and I am careful when I go out there.” Despite taking all precautions, P4 said; “I am stressed and afraid of catching it [coronavirus]”. Another participant P7 continued; “Well with COVID, you do not know where it is safe, and we are at risk. If I were to get the virus it would probably kill me. So, we wear masks and face shields, and we social distance. We went to the grocery store once; we will never do it again. I had difficulty breathing with the mask. Now, we do our shopping online.”

Participants mentioned having difficulties scheduling their appointments during the pandemic, where participant P5 stated: “Every 6 months approximately, but with COVID, my May appointment was cancelled, and I just spoke to him [pneumologist] on the phone last week.” She further recounted; “Before COVID I was supposed to have a one-on-one with a [COPD clinic] nurse, who would teach me how to cough more effectively, but with COVID nothing is happening.” Participant P3 underlined; “Follow-up care is rather restricted with COVID, it is not helping us.” Moreover, participants were afraid of the risks involved with going into the clinic during COVID-19. One participant P10 was unable to find transportation to the clinic; saying “I have had appointments that have been cancelled because of COVID. I was supposed to go in for a CT scan but in these times, I cannot get anybody to take me there. No one wants to take me to the hospital”. Participants resorted to telephone consultations with their pneumologists as they did not really trust going to the hospital. When discussing those tele-consultations, participants expressed concerns regarding the absence of physical examination. Participant P3 stated; “That is the thing that is useless because to get a proper evaluation of my oxygenation and lungs, you cannot do that over the phone. I would need to be on site and that is the problem right now.” Participant P5 added; “Well for the lungs it is a little strange because he [physician] cannot check anything, but it is almost a waste of time, although I presume if I was having more difficulties, then he would be able to deal with it […]. Phone appointments are a little more difficult.” This led participant P2 viewing tele-consultations as having to “self-diagnose” in lieu of the doctor; “I feel like sometimes I am diagnosing myself because everything is at a distance now.”

### Knowing someone is there to help, especially in COVID times

Throughout the interviews, it became clear that the telenursing services offered were helping in resolving much of the issues faced due to the conventional in-person follow-up care. When discussing the attention provided by the telenurse, participant P5 stated; “So, I see her [telenurse] as our monitor, just making sure that everything is fine.” Many participants (P1, P3–4, P6–7, P8, P10) were contacting the telenurse or were being contacted by her once or twice a week, particularly when they did not feel well. Although two participants (P2, P4) were unaware prior to the interview that they could contact the telenurse themselves, they were excited at the future prospect after being informed. When indicating on the questionnaires that they were not feeling well or had pain, participants P2 and P4 mentioned receiving a phone call from the telenurse promptly. In fact, the knowledge that their clinical data was being read was reassuring to most participants (P1–8). Participant P2 noted; “I was wondering if this is going off into the Internet, and nobody is reading it or what is happening with it. But I realized that she [telenurse] is definitely reading it after she called me.” Participants even found the conversations with the telenurse pleasurable and reassuring. Participant P3 mentioned; “it is a pleasure talking to her, she is very pleasant to know. And from my end, it is comforting to know that the data I put in is being used, somebody is looking at it, which is good.” Furthermore, the telenurse integrates personalized follow-up care, incorporating prophylactic nursing care tailored to participant needs, and facilitates participant empowerment with COPD management. For example, participants P2 and P4 were urged by the telenurse to take a COVID test based on their indicated symptoms or provided medication education. Others (P6–7) were advised to get their regular vaccines, including their pneumonia shot; “actually she [telenurse] was the one that told us about the pneumonia shot. She also asked me if I was allergic to any medication.” Others still, such as participant P8 were notified of possible environmental factors involved with COPD related symptoms they were experiencing. Participant P8 commented on the follow-up provided by the telenurse; “I am happy to do this this way, it is very simple […] the nurse is very friendly too. I talked to her a few times, she made me notice that the humidity levels in my house were too high, and that I needed to leave the air conditioning on more. She gave me very good advice.” Participant P1 recognized that telemonitoring had been an emotional relief: “It’s good to know someone is there to help, especially with the [corona] virus.”

Overall, participants found that the telenurse provided receptive and reliable feedback. So much so that participants (P1, P7–8) were emailing copies of changing or new prescriptions and health reports to the telenurse to ensure that she was up to date with their condition. Participant P1 stated: “I sent an email to her [telenurse] regarding my medication list and my nodules in my stomach and my throat.” In fact, most participants (P1–2, P4–5, P6–7) felt that given the current COVID context and the increased isolation that is necessary to protect themselves, a video phone call with the telenurse would be a great addition to this feedback and improve the telenurse’s ability to assess participant condition and body language. Participant P4 stated; “It would be very interesting. Because I do not know what Madame [telenurse] looks like, I would love to put a face to the communication” demonstrating the openness to using videoconferencing. However, two participants (P3, P9) felt that videoconferencing was unnecessary, and a telephone consult was sufficient for their follow-up. Participants (P1–3, P5, P9–10) also felt additional feedback from the telenurse was unnecessary due to the amount of contact they already had with her, as participant P9 stated; “No, because if I needed more things, there is a place below where you can ask to have a videoconference with her [telenurse]. I think that is perfectly okay.”

Another component that participants felt was more personalized in their follow-up care were the daily and weekly questionnaires. Regarding the daily questionnaire, all participants found it to be short and quick, and they answered the five questions roughly at the same time, mostly to form a habit. One participant P8 used a sticky note on the screen as a reminder to complete the questionnaire and another P2 wrote it in her agenda. All participants were very committed to filling out the daily questionnaire, the couple (P6–7) even getting out of bed once they remembered they had forgotten, to fill out the questionnaire. Some participants found the daily questionnaires dull and repetitive (P5, P6–7) although they understood the need to ask the questions; “I mean, it gets too boring. ‘Oh god, same question again and again’. But I can understand why you need to ask those questions” (P5). Many participants (P1–2, P4, P8–9) were comfortable filling the questionnaire out every day for the project period. Participant P2 stated, “My first thoughts were ‘Good God. Twelve months? I do not know if I can do this for twelve months’ […]. I think it is alright. It is good to know someone is there to help, especially with the COVID-19 virus.” In terms of the weekly questionnaire, participants’ opinions varied. Some participants (P2, P4, P8–9) did not mind completing the weekly questionnaire, saying “it is really not long” (P8), and “It is perfect, I have my whole life ahead of me” (P4), while participant P5 indicated; “Sometimes I feel like it is a waste of time, especially with the weekly ones, because I answer the same thing every week […]. I do not think it [weekly questionnaire] is necessary unless something came up.” Most participants (P1, P3, P5, P6–7) were eager to see more options and nuances in the questions regarding the weather and their symptoms to make the experience more personalized. Two participants (P4, P9) felt they needed more space to include comments. Although others (P2, P3–4) felt that more space would often lead to a timely phone call from the telenurse to follow-up on participants expressed concerns. Participant P3 stated; “Whenever I write it in the comment section, I know I most likely will get a call from the nurse.” Furthermore, participants P3 and P5 used the comment section to better identify their symptoms and include environmental and contextual details they thought might be relevant to their care, fostering self-management and independence. For example, participant P5 stated; “I put in that I had my grandsons over, because I figured that could impact my daily values.” Other participants P1 used the comment section as a space to provide a summary of the times daily they were not feeling well. Overtime many participants (P1–2, P4, P9) also became able to recognize their exacerbation symptoms. Participant P4 stated their primary symptom associated with an exacerbation is “abnormal breathlessness.” They were then establishing relationships between these symptoms and the environment, using these relationships as health indicators to better understand and gain a sense of control over their COPD. Overall, participants felt that having a telemonitoring system accompanied by the constant attention of a telenurse made them feel special, at ease, and allowed them to be less anxious during the challenging times of COVID-19.

### Unparalleled benefits of an oximeter

Astonishingly, despite the chronic nature of COPD, most participants (P1, P4–6, P8–10) expressed the lack of prior education they had received on the subject and had limited understanding of their condition. Participant P5 stated; “I have none. I am not even completely sure what it is. I mean I realize it is because I am smoking, and still smoking, and I get shortness of breath and have trouble catching my breath, and cough a lot, but thankfully I have not needed to go to the hospital. But that is about it.” Most participants (P1–2, P4, P7, P10) expressed that their prior education on COPD took place years ago, for some (P7) over 10 years ago, often at the time of their diagnosis with no follow-up education provided. This education was always by nurses, and over the years, many questions remain unanswered. Participant P6 stated that her education about COPD came from her husband who was also diagnosed with the same condition; “My husband tells me all about it.” As a result, participants (P1, P6–7, P9) began to actively seek information on the Internet. Participants P3, P8, and P10 were not interested in any information and felt that their knowledge was sufficient or that they would not benefit from any further information. Participant P4, who was directed to the COPD resources on the telemonitoring platform, found it “wonderful”, and stated further, “receiving education would take away panic and fear, and I could make connections [with the cause of the breathing problems], specialists often will not think about giving information.” Overall, most participants (P1–2, P4–5, P6–7) were eager to learn about their COPD, such as breathing exercises that may better enable them to cope with the symptoms associated with their chronic illness, despite lacking education and resources since their diagnosis.

Interestingly, what is more important is that despite a lack of education and limitations faced due to the COPD, participants (P1–2, P5, P6–7) were regularly noting down their pulse and oxygen saturation to see trends in their own data. Participant P5 mentioned; “I was actually starting to write down what I had recorded the day before, so I can compare my values daily.” Many participants (P2, P4, P6–7, P9) were monitoring their saturation and pulse parameters multiple times a day. Participant P7 stated that the oximeter was his “crutch”: “When I do the floors and dishes. I sit down on the couch, and I put it [oximeter] because it calms me down and I tell myself ‘do your breathing and it works’ […] it is my crutch.” Another participant P2 used their oximeter as a motivator to increase their oxygen saturation; “It’s a fantastic tool, and it tells me ‘Okay, you have got to bring this up’ or ‘If you are doing this, your oxygen drops down to 85’.” Still, other participants P3–4 used it multiple times only when they did not feel well or felt shortness of breath; “I use it often if I do not feel well. I learned how to breathe last week at the hospital, so I always do my breathing beforehand to make sure that I am above 90%” (P4). Participant P8 uses the oximeter as a means of reassurance; it is reassuring, when I do not feel as well, when I am tired. Then I take it, and often my oxygen is above 91% and I tell myself that I am alright, I do not have to worry.” In fact, some participants (P1–3, P7) currently have their own oximeter in addition to the project oximeter and two participants (P4, P8) were interested in buying an oximeter once the project finishes to continue to monitor their oxygenation and pulse. When asked, most participants (P1–2, P4–5, P6–7, P8, P10) were willing to submit these values multiple times a day (including after activities) and in multiple different positions (sitting, standing, lying down), although currently only took it sitting, often after calming down. Participant P1 stated: “This [oximeter], I think is marvelous, because with your project, participants take their levels standing, sitting, and walking […] this way, you can compare the progression in a month.” Similarly, participant P10 was eager to see how the temperature would affect the trends in their pulse and saturation, and participants P6–7 wanted to monitor pulse trends for other conditions besides COPD.

### Fragmented levels of care to integrated healthcare delivery

Communication among healthcare providers plays an important role in facilitating the continuity of care for COPD patients, however, when asked whether participant’s general practitioner and pneumologist communicated, P10 answered: “I do not know if they have ever been in contact with each other.” Some participants (P4, P8) did express interest in opening a line of communication between different providers and the benefits it would bring to their follow-up care plan; “If I have a problem with my lungs, I think it is important to start on the base level with my family doctor, to get to the pneumologist, but that these two people be linked so that my family doctor is aware of what is going on with my lungs. I really like communication systems. If they talk to each other, my God all would go well.” Most participants (P2, P5, P6–7, P10) expressed that having their general practitioner on a telemonitoring system would be valuable. Participant P5 remarked; “If my general practitioner could have access to my information, I think that is very important. Back in the day the general practitioner was your post office, it’s where all the information came back to and nowadays, they do not get anything. They are the hub.” Another participant P10 continued; “Any of the doctors I have to deal with should have access to what has been going on. If I need treatment, they have a file to look at. They do not need to go through all sorts of unnecessary tests to find out what is wrong. They will know what medications you are on so they will not give you the wrong medication. I think information between doctors should be shared.” Thus, telemonitoring was thought to be a valuable resource in facilitating the continuity of care for COPD patients. For some participants (P6–7, P10), it was unclear whether their physician would be interested in, or had even heard of, the possibility of a telemonitoring system where information can be shared. The couple (P6–7) was concerned regarding their general practitioner’s availability; “Our family doctor, that would be good […] but I do not think she got everything online […] her being involved would be great. But I do not know if she would have the time.” Another participant P8 mentioned the lack of digital technologies in her family physician’s office; “There is no computer in his office. Everything is done by hand, so there is not even a machine to measure oxygen levels. He is an old-fashioned doctor, there are no computers, just a fax at his desk. For him, it would be impossible to join this telemonitoring project.” She continued; “I think that it would become a bit heavy for him [family physician], I do not know. Maybe it would be interesting for another doctor that has the informatics in his office. But for now, he [family physician] receives my written reports.”

Overall, once the participants became acquainted with the telemonitoring system, they began understanding its importance and effectiveness in providing integrated follow-up care. Participant P5 mentioned; “Right now this is a survey and only for research, but if this [tele-monitoring] is available for usage, I see this is being extremely helpful. I just see this as another possibility in reducing people waiting in the emergency rooms […] I think it would be super.” Participant P4 explained; “The goal of the platform is to integrate different levels of care and I am in full accord with that.” Participant P9 even expressed that the system provides him with support and confidence, stating; “For me, it helps me understand and then I can talk about it with the telenurse. At least I have support. Before, I did not have support. If I was afraid or anything, I had to go to the emergency.” When discussing the system, participant P9 even stated the benefits of the system for the healthcare system as a whole; “That is what I think is important, for the community, for everything, for the hospital system, for our safety.”

### Proving misconceptions otherwise

Although the use of telemonitoring has increased, it can still be perceived as providing impersonal healthcare whilst being associated with a lack of privacy and confidentiality of participant information. However, in this study, all participants expressed feeling secure providing their health information on the platform. Participant P5 expressed; “I am fine with [information exchange through the platform] as long as it remains confidential.” Participant P10 even signified; “I don’t think many people will be interested in my heart rate.” She further elaborated; “I think you guys have thought it out quite well before asking the public to report their information.” Moreover, when asked about any privacy concerns sending their information, participant P3 replied; “Oh, no way … no, there is nothing I do not want to share or be ashamed of … no I have no hesitation.” Ultimately, participants trusted the researchers and platform developers to have created a safe place for their data to exist and remain confidential. One of the participants, P4, even recognized the value of the program and mentioned the importance of sharing their confidential information for their own health benefit; “I am very comfortable with everyone having access to my information because it is better for my health.” Similar comments were made by participant P8–9, where participant P8 stated; “My mind is very at ease with that [sharing information].”

Another preconception regarding the implementation of telemonitoring is the ability of participants to easily adopt technology and navigate a digital health platform. In this study, most participants (P1–3, P6–7, P10) were comfortable using technology: navigating the platform, inputting their data, sending, and receiving emails, and handling the project equipment. Only two participants (P2, P5) expressed some glitches at the start; “No, no. I did not find it [using the platform] difficult at all. There have been little hiccups but the [research assistant] got me through [ …]” (P2). In the beginning, participant P5 had some challenges accessing the platform on her mobile device and operating the platform in general but stated; “I was having problems for a while, until I figured out how to save the link to the portal. But since then, nope.” All participants expressed appreciation for the training provided on how to use the platform and felt that it was sufficient. Participant P3 elaborated; “Went through it, repeated it so no it was clear as a bell. It was not that complicated.” Another participant P10 stated; “No, I do not think so” when asked whether any additional training would have been beneficial, and that she understood “where she was supposed to go” on the platform. Overall, participants displayed interest in the project and demonstrated so by consistently asking questions about how they could improve their own self-monitoring and showing interest in the project’s success. Participants were curious about when and how they should take their vital signs and learning more about COPD. One participant P5 expressed genuine interest in which factors contributed to her irregular symptoms: “I know my breathing capacity changes, according to the humidity and temperature. Would it not make more sense for me to put my vital signs and the temperature [weather]? Since the weather changes throughout the day.” Not only did participants show interest in their condition, they were also committed to their role and success of the telemonitoring project.

## Discussion

The COVID-19 pandemic has been challenging, however, for patients with a chronic condition such as COPD, who are at a higher risk for being infected with the coronavirus, this period has been especially difficult due to the lack of continuity in their follow-up care. On several occasions, medical appointments have been cancelled, postponed, or were conducted over the phone. The provision of integrated telehealth nursing services was for all participants a welcome and pleasant alternative, and many saw the telemonitoring system as a mean to connect levels of care within our healthcare systems. Based on our findings, two points warrant discussion: 1) Participant’s attachment to their pulse oximeter as a barometer of their health; 2) Participant’s preferred follow-up plan for a long-term adaptation and sustainability of telemonitoring services.

As per the clinical protocols of the study, participants used a non-invasive pulse oximeter device as a method to measure their blood oxygen saturation in a matter of seconds. Normal peripheral oxygen saturation (SpO_2_) levels range between 95 and 100%; however, for patients with COPD, the target range is between 88 and 92% [18, 19]. Interestingly, the findings of this study indicated that participants were not only following the protocol and measuring their oxygen saturation once a day but tended to measure their levels multiple times a day, for example after physical activity and when they noticed changes in the weather. The majority used a personal threshold value for their oxygen saturation as a motivator and an indicator to gauge their health status, and form patterns to prevent COPD exacerbations. Providing our study participants with an oximeter enabled them to control and manage their condition, and as supported by other literature [20, 21], it improved their anxiety and provided them with a sense of empowerment. Although widely accepted in clinical and home settings, a pulse oximetry should never replace an arterial blood gas analysis and should only be used as a screening tool when low blood oxygen levels are suspected. The literature suggests physicians to limit the use of the pulse oximeter to acute events, for example, patients presenting with a COPD exacerbation, or for specific groups of patients only, and as a valuable addition to their regular clinical patient assessment [22]. A pulse oximetry may detect SpO_2_ deteriorations and trigger if needed a higher level of care, yet, COPD evidence-based guidelines suggest not to rely on a pulse oximeter only to determine how a person should be feeling [23]. It is perfectly possible that a person with COPD has normal SpO_2_ levels while feeling extremely short of breath and/or experiencing other troubling symptoms, and vice versa have unhealthy SpO_2_ levels, without any noticeable symptoms. Moreover, an exacerbation can have a very sudden and an idiopathic onset, which may or may not be correlated with a foreseeable change in the SpO_2_ levels of the patient [21, 24]. Thus, a continuous reliance for patients on an oximeter and a perceived personal threshold oxygen saturation may provide them with a false sense of security, which can be dangerous especially for those patients lacking proper COPD education. It is unfortunate, as according to our findings and current evidence, patients with COPD still express a need for more disease knowledge and self-management behaviors [21, 25–27].

Inadvertently, an increased popularity of wearable finger pulse oximeters and blood oxygen sensors incorporated in wearable commercial health devices is promoting COPD patients to increasingly purchase these devices to self-monitor their SpO_2_. Oximeters in commercial devices such as the Apple Watch 6 are not yet approved by the Food and Drug Administration (FDA) for clinical use and can overestimate the blood oxygen saturation in patients with COPD [28, 29]. Furthermore, when using commercial finger pulse oximeters, it is much more likely for black or brown skin patients to have occult hypoxia, where the pulse oximeter readings indicate a normal range whereas the arterial blood gases level is hypoxic [28]. Despite patient’s increased reliance on their oximeters for reassurance purposes, as according to our findings, the probability of false measurements may consequently create an increasing number of false SpO_2_ alerts monitored, which may result in unnecessary follow-up interventions by the telenurse. To provide appropriate remote follow-up care, it is imperative that the telenurse monitors a composite of patient’s heart rate and SpO2 to reduce false alarms whilst distinguishing exacerbation onset from symptom variation, and potentially facilitating prompt intervention, as both parameters are calculated from the same photoplethysmogram (PPG) signal as measured by oximeters [20, 24]. In addition, proper patient education and training is needed on the positioning of the pulse oximeter and the interpretation of a combination of SpO_2_ and heart rate [30], as well as reliable oximeters and standardized clinical protocols for telemonitoring services [30].

Overall, participants were excited in participating in this telemonitoring project and expressed an interest to continue beyond the project duration, except for one participant who was initially hesitant to enroll not knowing if she would be able to commit to submitting the requested questionnaires for the 12-month period. Other participants also remarked on several occasions that they were motivated to participate to enhance the success of the research, which raises a key question regarding the willingness of patients with COPD to adopt a long-term use of telehealth nursing services. Currently, little evidence exists regarding the compliance and adherence of COPD patients to the usage of long-term telemonitoring services. In contrast to heart failure patients, COPD patients’ rate and duration of hospitalizations did not improve over a 4-year period when using an integrated at home telemonitoring service [31]. Moreover, literature on telemonitoring services for various chronic patient populations indicates a low adherence rate to the protocols and guidelines over a long period of time with an average decline in response frequency to follow-up questions of 1.4% per month [32]. In our study, participants expressed a lack of interest in answering the daily and weekly questionnaires when their symptoms did not defer from the prior days, and in essence they found the questionnaires redundant. In addition, participants’ opinions regarding the use of the comment section were divided; some would write extensively, while others hesitated as it would lead to an immediate follow-up call from the telenurse. The extensiveness of the questionnaires and its response frequency of patients warrant discussion, however, evidence exists that patients using telemonitoring services prefer a 2 to 3 day per week response rate [33, 34]; allowing for increased autonomy and adherence to lifelong chronic disease management. Although patient preferences must be considered when standardizing the clinical telemonitoring protocols, several participants expressed no reluctance and instead encouraged sharing their personal data with the clinical team across levels of care. This information sharing among providers would enhance patient coordination of care, however, it raises the issue of interoperability among healthcare systems. If a telemonitoring system would be incorporated into an electronic health record [35], it would promote a more balanced approach between patient preferences in terms of follow-up response frequency and the clinical data needed for a telenurse to provide appropriate interventions. Further research is needed on the aspect of preferred patient follow-up plan and the sustainability of telehealth nursing services in an integrated healthcare system.

## Limitations

This study poses the following limitations and opportunity. The first limitation is geared towards the context in which the data collection took place. Interviewing participants 3-months after the project initiation during the initial lockdown phases of the pandemic might have shed a different light on aspects of long-term adherence to the telemonitoring service and further details regarding patients’ preferences, in comparison to the completion of the project. Second, our study participants suffered from varying levels of COPD severity and other comorbidities, which may have impacted their views and willingness to adhere to the clinical protocol in terms of using their oximeter and answering the daily and weekly questionnaires. Finally, despite the study receiving ethics approval in 2019, the project was postponed due to platform-related technical challenges and as such happened to take place during the COVID-19 pandemic. Although not a limitation, this change in implementation date created an opportunity to explore the clinical protocol and set-up of the telehealth nursing services during extraordinary circumstances.

## Conclusion

Although telemonitoring is effective for patients with COPD, this study demonstrated that the provision of integrated telehealth nursing services during a pandemic is a promising strategy to provide continuous and responsive follow-up care in our existing fragmented healthcare delivery system. In general, due to the support from the telenurse, participants expressed feeling empowered, having improved anxiety, and the ability to self-manage their condition on a day-to-day basis. However, the overreliance on the use of the project provided oximeter and the diffuse opinions of participants regarding the questionnaires, triggered the need to conduct further research on patient preferences, the standardization of protocols, and the integration of telehealth nursing services within the healthcare system. Further exploration of these aspects would be imperative for a sustainable long-term adaptation of integrated telemonitoring services.

## Data Availability

The datasets generated and/or analysed during the current study are not publicly available due to the explicit assurances of the authors to participants that their confidentiality would be respected, but are available from the corresponding author on reasonable request.
